# Improved Local and Systemic Anti-Tumor Efficacy for Irreversible Electroporation in Immunocompetent versus Immunodeficient Mice

**DOI:** 10.1371/journal.pone.0064559

**Published:** 2013-05-24

**Authors:** Robert E. Neal, John H. Rossmeisl, John L. Robertson, Christopher B. Arena, Erica M. Davis, Ravi N. Singh, Jonathan Stallings, Rafael V. Davalos

**Affiliations:** 1 Department of Radiology, The Alfred Hospital, Melbourne, Victoria, Australia; 2 Bioelectromechanical Systems Laboratory, Virginia Tech-Wake Forest School of Biomedical Engineering and Sciences, Virginia Tech, Blacksburg, Virginia, United States of America; 3 Department of Small Animal Clinical Sciences, VA-MD Regional College of Veterinary Medicine, Virginia Tech, Blacksburg, Virginia, United States of America; 4 Center for Comparative Oncology, VA-MD Regional College of Veterinary Medicine, Virginia Tech, Blacksburg, Virginia, United States of America; 5 Cancer Biology, Wake Forest University Baptist Medical Center, Winston-Salem, North Carolina, United States of America; 6 Department of Statistics, Virginia Tech, Blacksburg, Virginia, United States of America; University of California at Berkeley, United States of America

## Abstract

Irreversible electroporation (IRE) is a non-thermal focal ablation technique that uses a series of brief but intense electric pulses delivered into a targeted region of tissue, killing the cells by irrecoverably disrupting cellular membrane integrity. This study investigates if there is an improved local anti-tumor response in immunocompetent (IC) BALB/c versus immunodeficient (ID) nude mice, including the potential for a systemic protective effect against rechallenge. Subcutaneous murine renal carcinoma tumors were treated with an IRE pulsing protocol that used 60% of the predicted voltage required to invoke complete regressions in the ID mice. Tumors were followed for 34 days following treatment for 11 treated mice from each strain, and 7 controls from each strain. Mouse survival based on tumor burden and the progression-free disease period was substantially longer in the treated IC mice relative to the treated ID mice and sham controls for both strains. Treated IC mice were rechallenged with the same cell line 18 days after treatment, where growth of the second tumors was shown to be significantly reduced or prevented entirely. There was robust CD3^+^ cell infiltration in some treated BALB/C mice, with immunocytes focused at the transition between viable and dead tumor. There was no difference in the low immunocyte presence for untreated tumors, nude mice, and matrigel-only injections in both strains. These findings suggest IRE therapy may have greater therapeutic efficacy in immunocompetent patients than what has been suggested by immunodeficient models, and that IRE may invoke a systemic response beyond the targeted ablation region.

## Introduction

Focal ablative therapies are an important alternative to traditional surgical excision in clinical oncology. Many of these therapies utilize thermally-mediated tissue effects (heating or cooling) to kill the targeted volumes of cells, such as radiofrequency ablation, cryoablation, and high intensity focused ultrasound [1]–[3]. In addition, non-thermal therapies such as electrochemotherapy (ECT), electrogenetransfer (EGT), and irreversible electroporation (IRE) are gaining momentum as viable non-thermal therapeutic approaches to tumor therapy and focal ablation [4]–[7]. Such therapies offer a number of advantages compared to thermal techniques because the non-thermal nature of electroporation pulses, including IRE pulses to induce localized cell death, spares the extracellular matrix, major vasculature, and other sensitive structures [6], [8]–[10]. This facilitates rapid healing, minimizes scarring, and enables treatments to be performed on targeted regions within and adjacent to sensitive structures, permitting treatment of tumors unsuitable for surgical resection or thermal therapies [6], [8].

IRE procedures involve placing small (∼1 mm diameter) needle electrodes into the targeted region to deliver a series of brief (∼100 µs long) electric pulses. These pulses alter the cellular transmembrane potential, resulting in irrecoverable membrane defects that kill the tumor’s cells in a controllable manner with sub-millimeter demarcation between treated and normal regions [10]. Treatments can be planned with numerical modeling [11], may be administered quickly (∼5 minutes), and are not significantly influenced by blood flow “heat sink” effects [12], [13]. IRE ablation requires no chemicals or adjuvants, and has achieved promising complete regressions in experimental murine tumors [14], [15] as well as veterinary case studies in complex, otherwise inoperable tumors [5], [16]. Human phase I clinical trials have obtained complete tumor remission for 46 of 69 total renal, liver, and lung tumors (66%) unresponsive to or ineligible for current standards of care [6], with promising clinical outcomes for additional soft tissue tumors, such as pancreatic [17], [18].

Most ablation requires inserting a probe directly into the tumor, and the therapies are regarded as therapeutic only within the targeted region. However, since the tissue remains *in situ* following treatment, there is a release of antigens and the various signals of cellular distress (so called “danger” signals) in the treated and peripheral volumes that may promote local and systemic immune responses [19]–[21]. Observations of shrinking or regressing distant metastatic lesions in patients following thermally-based focal tumor treatments appear to indicate systemic anticancer responses [22], [23].

Further studies have combined focal ablation with adjuvant immunomodulating materials experimentally and clinically to augment the systemic effects observed in ablation therapies [24], [25]. Experimental murine tumors treated with a combination of ECT and injections of CpG oligodeoxynucleotide [26] or modified IL-2 secreting cells [27] were able to attain regressions in contralateral tumors from the same cell line, despite the inability to attain a significant distant antitumor effect in animal or human models with ECT alone [27]. The strength of immunomodulating materials is further conveyed by electroporation-vector *in vivo* gene-based immunotherapy, which has obtained local and distant protective effects experimentally and clinically using cytokine encoding plasmids [28], [29]. Such EGT-based approaches have been shown to work synergistically with ECT [30].

The ability for IRE to kill cells through a non-thermal mechanism may release substantial intact antigens and stress signals from the affected cells, resulting in an immune response that enhances therapeutic outcome locally and distant from the treated region. Although IRE-induced cell death has been shown to occur independently from an immune response, immunocyte infiltration and lymphatic drainage have been observed following tissue ablations *in vivo* [8], [12], [15]. In addition, an immunocompetent canine patient treated with IRE showed an affected tumor volume greater than predictions based on immunodeficient and healthy tissue experiments [5]. This contrasts with a study where tumor xenografts on immunodeficient athymic nude mice did not regress when the tumors were a few millimeters larger than the predicted treatment volume [14]. It is possible that the lack of an adaptive immune response in the nude animals may be responsible for this difference, suggesting immunodeficient athymic mice underestimate the efficacy of IRE. Finally, changes to immune markers were observed from tumors treated with IRE in immunocompetent rats, including changes in CD4^+^/CD8^+^ ratio, cytokine IFN-γ and IL-4-positive splenocytes, and serum sIL-2R and IL-10 [31], though the immune marker changes were not directly examined for their potential role in improving IRE therapeutic outcome. Based on these findings, we hypothesize that IRE anti-tumor efficacy should be stronger in immunocompetent subjects than those lacking a complete immune system, and that there may be a systemic benefit to distant tumors from IRE therapy.

To determine the potential effect of a functioning immune system on IRE tumor treatment outcomes, we performed identical IRE treatments on immunodeficient (ID) athymic (nude) mice and immunocompetent (IC) BALB/c mice bearing subcutaneous murine renal carcinoma tumors. In order to delineate any difference in treatment outcome between the two mouse groups, we treated the tumors with an IRE pulsing protocol that used a voltage-to-distance ratio 40% lower than one that obtained a 92% regression rate in ID mice [15]. Treatment response was evaluated by tumor growth, histological assessment, and rechallenge.

## Methods

### Ethics Statement

This study was carried out in strict accordance with the recommendations in the Guide for the Care and Use of Laboratory Animals of the National Institutes of Health. The protocol was approved by the Virginia Tech institutional animal care and use committee (IACUC, protocol number 11-019-SBES). All surgery was performed under isofluorane anesthesia followed by buprenorphine analgesic, and all efforts were made to minimize suffering.

### Mice

Two strains of female mice, immunodeficient (ID) Nu/Nu athymic nude and immunocompetent (IC) BALB/c, (Charles River Laboratories, New York, NY) were housed in individually ventilated cages in groups of five under specific pathogen free conditions. Mice were allowed access to sterilized water and feed *ad libitum*, and were kept in the animal facility for approximately 2 weeks, reaching an age of 7–12 weeks and weighing 15–25 g prior to tumor implantation.

### Tumor Implantation

Renca murine kidney cancer cells (CRL-2947) were obtained from the ATCC (Manassas, VA) and verified by Short Tandem Repeat DNA profiling. The Renca cell line was derived from a spontaneous renal cortical adenocarcinoma in BALB/cCr mice as previously described [32]. Cells were cultured in RPMI 1640 media supplemented with 10% fetal bovine serum, 1% penicillin/streptomycin, and 1% sodium pyruvate (Fisher Scientific, Waltham, MA). Cells re-established from frozen stocks were expanded for two passages then frozen again and stored in liquid nitrogen. Prior to tumor implantation, these cells were thawed and expanded for three passages. For all studies, cells were maintained in continuous culture for no longer than three months.

Once reaching approximately 80% confluence, cells were twice washed with phosphate buffered saline (PBS) and treated with 0.25% trypsin EDTA (Fisher) for detachment from culture flasks. Cells were resuspended at a concentration of 1×10^7^ cells/ml in a 50/50 mixture of PBS and Matrigel High Concentration (BD Biosciences, San Jose, CA). Mice were anesthetized by inhalation of 3% isoflourane (Abbott Laboratories, Abbott Park, IL) for induction and 2% for maintenance. The hair on the BALB/c mice was clipped at the injection site, and the flank of both mice strains was sterilized with isopropyl alcohol prior to injecting 200 µl aliquots of the cell suspension (2×10^6^ cells total) subcutaneously on the rear right flank of 18 nude and 18 BALB/c mice. An additional 8 mice from each strain received 200 µl of the matrigel and PBS mixture only, without any tumor cells, in order to histologically evaluate baseline immunocyte response to the inoculation matrix.

### Tumor Treatment

Tumor growth was measured using calipers. Once reaching a minimum of 5×5 mm in top cross-sectional area and approximately 4 mm deep, tumors were treated in 11 mice from each strain. Mice were anesthetized following the same isofluorane inhalation protocol from tumor implantation. The skin over the tumor was prepped with 70% isopropyl alcohol, and 2 insulated metal needles with 1 mm exposed length were inserted into the tumor to measure the voltage drop between them to ensure effective delivery of the electric pulses to the tumors. The needles were 0.3 mm in diameter and separated by 1 mm center-to-center. A layer of conductive gel (4.15 S/m) (Parker Laboratories, Enumclaw, WA) was applied to opposing ends of the tumor, perpendicular to needle orientation. In addition, a thin layer of gel was applied to the inward-facing plate portions on custom-built electrodes made from a modified BTX Caliper Electrode (Harvard Apparatus, Cambridge, MA). The electrode was modified to use custom-built plates contoured to the mouse’s body, providing a larger surface area for pulse delivery, and which were insulated on the non-contact regions using a thin layer of epoxy.

**Figure 1:**
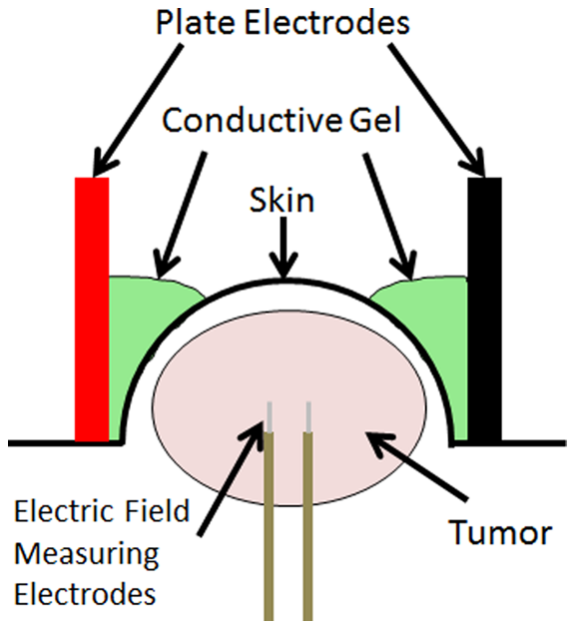
Schematic of experimental setup. Plate electrodes are placed on either side of the subcutaneous murine tumor, with a highly conductive gel facilitating improved current delivery into the tumor. Two small electrodes are inserted directly into the tumor perpendicular to the plate electrodes to measure the voltage drop between them, ensuring adequate electric field distribution through the center of the tumor. Treated groups had IRE electric pulses delivered through the plate electrodes, while controls had no pulse delivery.

The custom-built pulse delivery plate electrodes were placed on opposing sides of the tumor, perpendicular to needle orientation (Fig. 1), and tightened until producing minor compression of the tumor. Plate separation was measured, and the ECM 830 square wave pulse generator (Harvard Apparatus, Cambridge, MA) was used to deliver electric pulses between the plates with a voltage-to-distance ratio of 1500 V/cm, each 100 µs long, delivered at a rate of 1 pulse per second. A total of 100 pulses were delivered, reversing polarity after the first 50. Following pulse delivery, the electrodes and needles were reoriented 90°, and the pulsing process was repeated, delivering a total of 200 pulses to the tumor. This protocol was selected due to its ability to produce an observable treatment response relative to controls for the tumors used, but not strong enough to cause complete regressions in both strains of mice, which would make it difficult to discriminate any differences in treatment outcome.

To ensure similar pulse delivery between the experimental groups, electrical current was measured using a Hall effect probe (Tektronix TCP 303) and amplifier (Tektronix TCPA300) interfaced with an oscilloscope (Tektronix TDS 1002B, Beaverton, OR). In addition, the needles inserted into the middle of the tumor were connected to an oscilloscope, where the electric potential difference between the electrodes was measured. By knowing the voltage drop and separation distance between the electrodes, we were able to calculate the delivered electric field to the tumor.

Seven mice with tumors from each strain received sham treatments once the tumors reached a treatable size, where the protocol setup was repeated without delivery of the electric pulses. Matrigel/PBS-only inoculations received pulsed treatments over the acellular Matrigel/PBS plugs. Following all procedures, topical antibiotic ointment was applied to the needle insertion wounds, mice were removed from anesthesia and given subcutaneous injections of 0.05mg/kg buprenorphine analgesic diluted in 2 ml sterile saline. All mice received analgesic daily for an additional 2 days.

### Tumor Monitoring and Reinoculation

Following observance of a visible tumor, tumors and weights were recorded three and two times weekly, respectively. Tumor volumes were calculated according to the equation for an ellipsoid, *V = 4/3·π·a·b^·2^*, where *a* is the long dimension of the tumor ellipsoid, and *b* is the dimension perpendicular to the axis along *a*.

Mice were re-challenged with a second inoculation in their contralateral left flank 18 days following treatment. These mice were followed for an additional 15–16 days after reinoculation unless either the primary or rechallenge tumor reached a diameter of 18 mm in any dimension, after which they were euthanized. Matrigel/PBS-only inoculations of each mouse strain were euthanized at 18 days (time prior to rechallenge, n = 4 per strain) and 34 days (final study endpoint, n = 4 per strain) after pulse delivery.

### Histology

Once the mice reached a point to require euthanasia, samples of any present tumor tissue were excised and sectioned for processing. Representative tissues were preserved in 10% neutral buffered formalin and embedded in paraffin. Formalin preserved paraffin embedded samples were sectioned and processed for histology using Haematoxylin and Eosin and anti-CD3 antibody (rabbit polyclonal ab828; 1∶50 dilution, Abcam Inc., Cambridge MA, USA) on a Ventana automated immunostainer (Discovery XT, Ventana Medical Systems, Inc.) using a fast red detection system. All photomicrographs were obtained with a Leitz Wetzlar Orthoplan microscope using a Nikon DS-Fi1 charge coupled digital camera (Nikon, Japan). Digital images were imported into Nikon Elements AR image analysis software (Nikon, Japan) and saved on a personal computer.

### Statistics

Tumor response was evaluated from individual tumor dimension measurements taken at least 3 times weekly. Due to the nature of treating the mice based on tumor burden rather than time post-inoculation, this resulted in some variation among which days were evaluated. Therefore, the most recent observation for each mouse was imputed until a new observation was recorded. Median tumor size was evaluated for comparison according to the four basic protocol groups of A: ID/Control, B: ID/Treated, C: IC/Control, and D: IC/Treatment. Two-way ANOVA and Mann-Whitney post-hoc tests were performed on the square root of the median tumor size to test the statistical significance of tumor size differences between the groups at days 10, 15, 20, and 25 post-treatment. In addition, the potential for IRE treatment to induce a systemic protective effect against rechallenge was evaluated using a Wilcoxon signed rank test. This test assessed the significance in time required for tumors to grow to a treatable size for the rechallenge inoculation compared to the initial inoculation.

## Results

Initial inoculations produced tumors of treatable size after an average of 9.4 days for the nude mice and 9.7 days for the BALB/c mice, reaching average tumor volumes of 1728±868 mm^3^ on the day of treatment. Treatment associated complications were limited to superficial skin necrosis induced by transcutaneous pulse delivery, with resulting skin wounds resolving within 1 week of treatment. Electric field exposure within the tumor of the treatment groups had median and quartile (1^st^/3^rd^) ranges of 839 V/cm (749/1185 V/cm) for the IC treated mice and 838 V/cm (479/1055 V/cm) for the ID mice, showing similar tumor exposure to IRE despite the clipped fur on the skin of IC mice. Most variation likely resulted from variable bowing of the needle electrodes that measured the drop in voltage upon insertion into the tumor. The measured current between the plate electrodes had median and quartile (1^st^/3^rd^) currents of 4.1 A (3.6/6.1 A) for the IC mice and 4.6 A (4.0/6.2 A) for the ID mice.

### Initial Tumor Response

**Figure 2:**
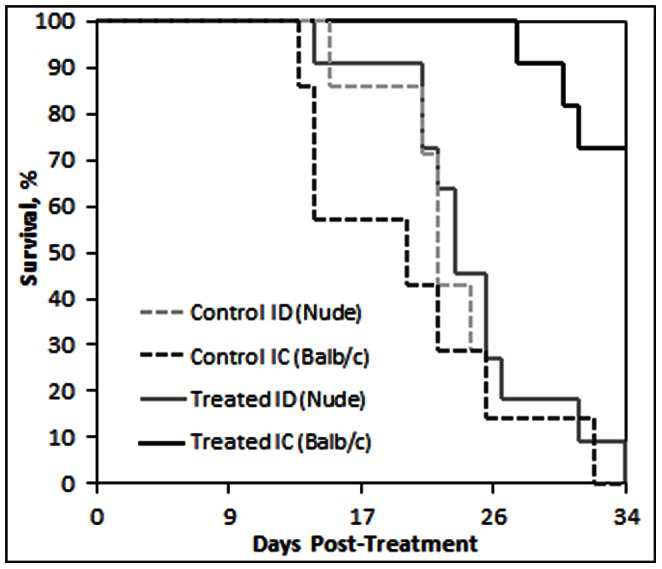
Kaplan-Meier survival plot. Survival of the different experimental groups based on euthanasia timepoints resulting from excessive tumor burden, when tumors reached 18 mm in any given dimension. The total experimental period was 34 days post-IRE treatment. Where 70% of the treated immunocompetent (IC) BALB/c mice survived until the endpoint of the study, nearly all control IC mice and both experimental immunodeficient (ID) nude mice groups were euthanized prior to reaching the endpoint due to excessive tumor burden.

A Kaplan-Meier plot (Fig. 2) shows the survival of the mice from all four treatment groups based upon their time of euthanasia due to tumors reaching 18 mm in any dimension, where greater survival is noted for the treated IC group.

**Figure 3:**
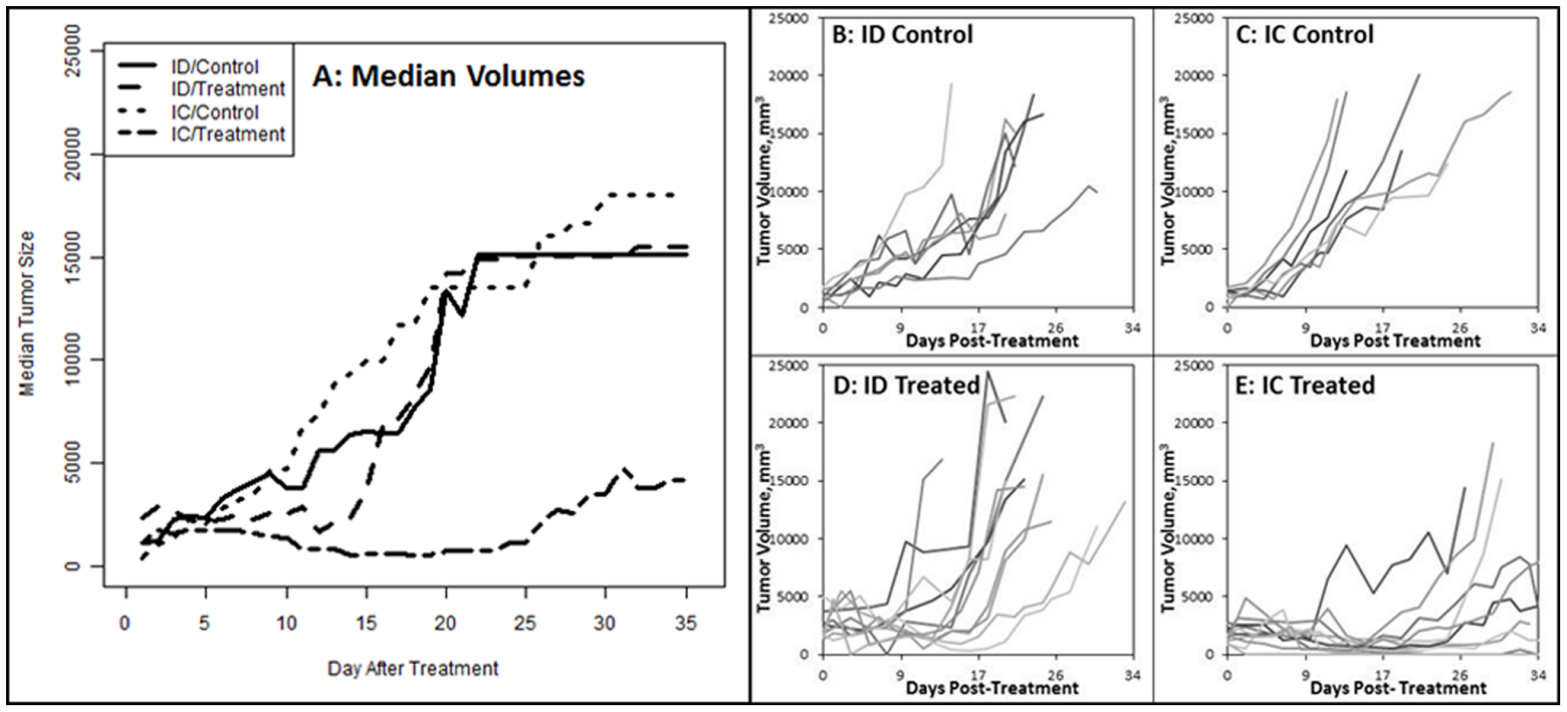
Original inoculation tumor volume curves. (A) Imputed median primary tumor volumes (mm^3^) for each group, showing significantly smaller median tumor size in the treated immunocompetent (IC) BALB/c mice. (B–E) Individual trial tumor comparisons between (B,D) immunodeficient (ID) nude and (C,E) immunocompetent mouse strains for (B,C) sham and (D,E) pulsed treatment groups, showing a clear improvement in progressive disease free survival for the treated IC mice (E) relative to both controls and treated ID mice (B–D). Treated ID mice show an initial pause in tumor volume for the first 12 days from the treatment, followed by progressive growth, a result of selecting a sub-optimal IRE pulsing protocol for this study. All endpoints in data are a result of euthanasia due to tumor reaching 18 mm in any dimension.

Imputed median (Fig. 3A) and individual (Fig. 3B–E) curves of initial tumor growth for both mouse strains and treatment groups shows that growth patterns for control groups from both mouse strains were similar. Treatment for the ID group initially delayed tumor growth, extending the period of progression-free disease before recovering to similar levels as both control groups. Treatment for the IC mice dramatically extended the period of progression-free disease relative to the IC sham controls. It is important to note that the imputation of data in Fig. 3A resulted in the carrying over of tumor dimensions from the time of euthanasia for mice killed prior to the 34 day endpoint due to tumor burden.

**Figure 4:**
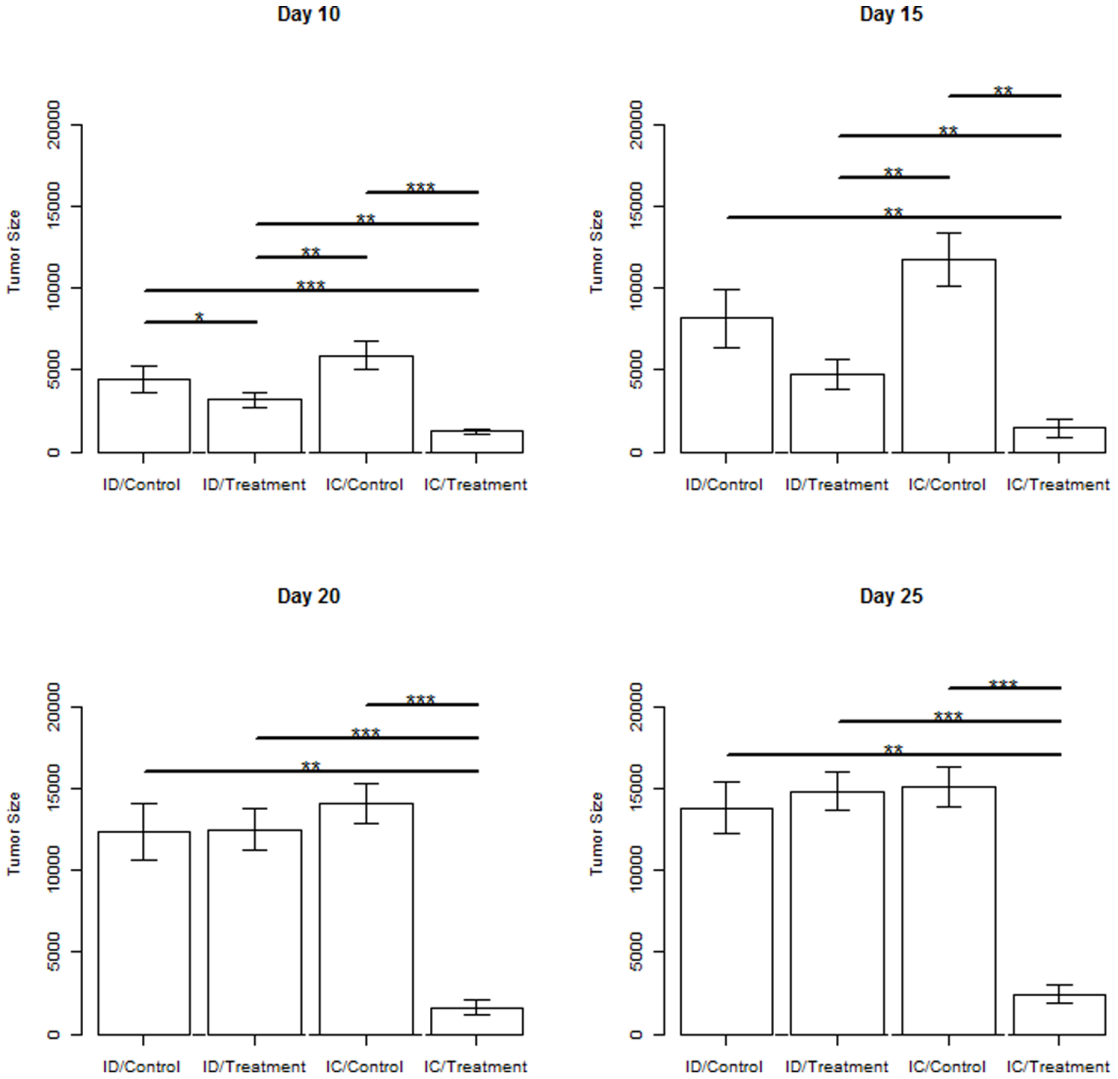
Primary tumor volume means and standard deviations. Mean tumor volume for all surviving mice at days 10, 15, 20, and 25 post-treatment. Mann-Whitney statistical significance was calculated between immunodeficient (ID) nude mice and immunocompetent (IC) mice that received treatment or sham control, showing statistically significant difference in mean tumor size for the groups at different timepoints, where treated ID mice show a difference at days 10 and 15, while treated IC mice show a significant difference for all time points considered. *0.05>p>0.01; **0.01>p>0.001; ***p<0.001.

Statistical analysis of differences in median and mean tumor volumes between the four treatment groups were carried out using two-way rank ANOVA (data not shown) and Mann-Whitney post-hoc tests (Fig. 4) at 10, 15, 20, and 25 days post-treatment. These tests confirm the qualitative assessments drawn from Figs. 2 and 3 to be statistically significant. From the ANOVA tests, it was shown that there is no significant difference (p>0.5) between all four treatment groups 5 days after treatment, but significance begins to appear between immune status and treatment starting at day 10. For the Mann-Whitney tests, the treated ID group was statistically different (p<0.05) from the untreated ID and IC groups at day 10, but is no longer different from either group by day 20. Furthermore, it is shown that the treated IC group is statistically different (p<0.01) from all three other treatment groups for all days examined beginning day 10.

### Tumor Rechallenge

Treatment group mice were rechallenged with a second tumor inoculation of the same cell line 18 days after IRE treatment. Only 5/11 ID mice were able to reach this reinoculation time point; with just 2/5 surviving 4 days beyond rechallenge without a tumor reaching 18 mm, and none reaching the full 16 days. Therefore, reinoculation data on the ID nude mice have been excluded from analysis.

**Figure 5:**
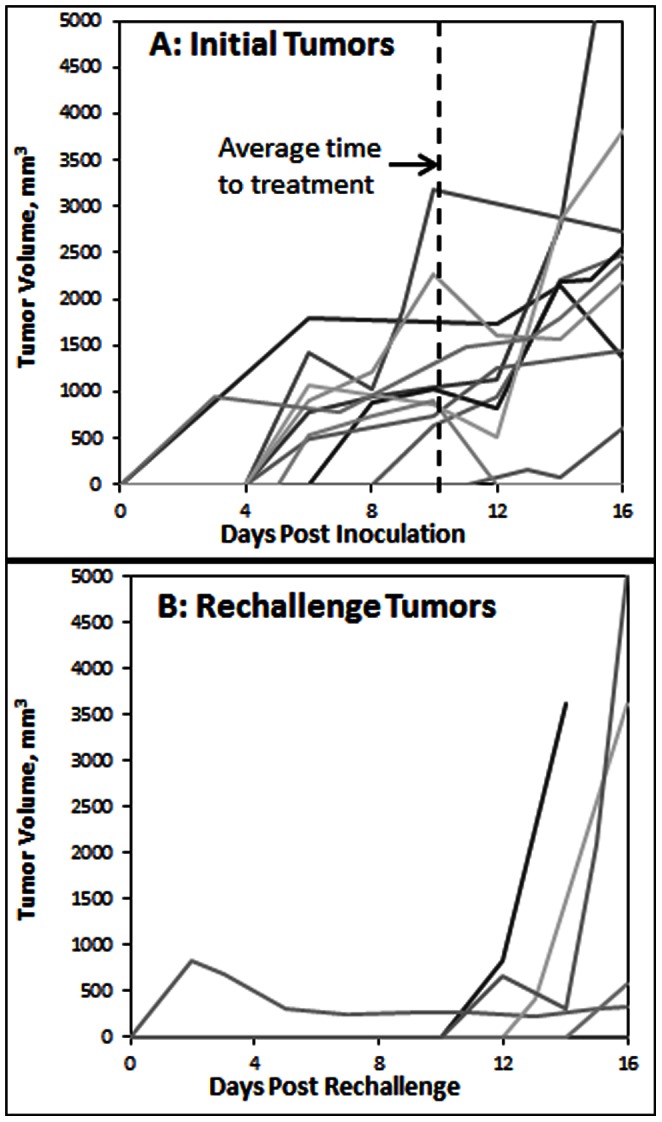
Primary and rechallenge BALB/c tumor growth curves. Growth curves for immunocompetent BALB/c mice for tumors grown after the (A) first inoculation and (B) rechallenge inoculation, which occurred 18 days after treatment of the first tumor. Average time for first inoculation tumors to reach minimum protocol treatment size of approximately 5×5 mm across and 4 mm deep was 9.7 days. Rechallenge inoculation tumors showed significantly delayed or completely inhibited growth of contralateral rechallenge tumors from the second inoculation.

Tumor growth curves for the original and rechallenge inoculations in the IC mice (Fig. 5) clearly show that the second tumor inoculations in the BALB/c mice had dramatically slower, if any, observable tumor growth. For the original inoculations, 10/11 tumors were observed to grow by 10 days after inoculation, with 10/11 reaching a treatable size within the 16 day period. Conversely, only 1/10 rechallenge tumors had grown by day 10, with only 3/10 reaching a treatable size over the 16 day period. Overall, there was no tumor recurrence at all in 5/10 mice, a protective effect of 50% over a period nearly twice that for the original tumors to reach a treatable size. It should be mentioned that while no measurable tumors were observed on 5/10 mice at all, there were 2 instances where no re-challenge tumor was observed over the skin during the 16 day rechallenge tumor period, but very small lobules at the rechallenge site were found when the skin was removed after animal euthanasia. When comparing Fig. 5A and 5B, it should be noted that the average time for the tumors to meet the treatment criterion was 9.7 days, so initial tumor growth data beyond this threshold should be considered as post-treatment volumes.

Statistical analysis of differential growth rates between the initial inoculations and rechallenge was performed using Wilcoxon one-sample test for the time required for the tumors to reach a volume of 700 mm^3^, the average volume prior to fulfilling treatment criterion. The test was performed on the smallest difference between the two observed time intervals for the tumors to reach 700 mm^3^, where the difference was found to be statistically significant (p = 0.0214).

### Histology

**Figure 6:**
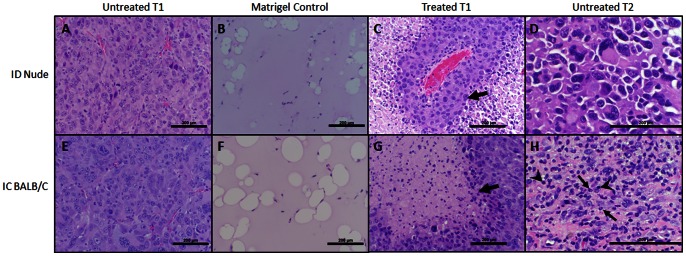
Haematoxylin and Eosin staining of various treatment groups. Histology of different inoculation conditions for (A–D) immunodeficient (ID) Nude mice and (E–H) immunocompetent (IC) BALB/C mice. (A,E) untreated initial tumors, (B,F) matrigel controls, (C,G), treated initial tumors (T1), and (D,H) untreated rechallenge tumors (T2). There is little overall immune reaction present in (A,B,E,F), the untreated T1 tumors and untreated matrigel controls, with no appreciable immunocyte presence difference between ID and IC groups. ID treated T1 (C) and untreated T2 (D) tumors had a relatively low immune response, with some neutrophils present. Arrows in (C,G) denote transition between dead and viable tumor cells in both group treated T1s. Treated IC T1 tumors (G) had most immune reaction at the transition zone between dead and viable tumor. Untreated T2 rechallenge tumors in the IC mice (H) show the presence of lymphocytes (arrowheads) and polymorphonuclear leukocytes (arrows). All scale bars 200 µm. Panels (A,B,C,E,F,G) 200x and (D, H) 400x magnification.

**Figure 7:**
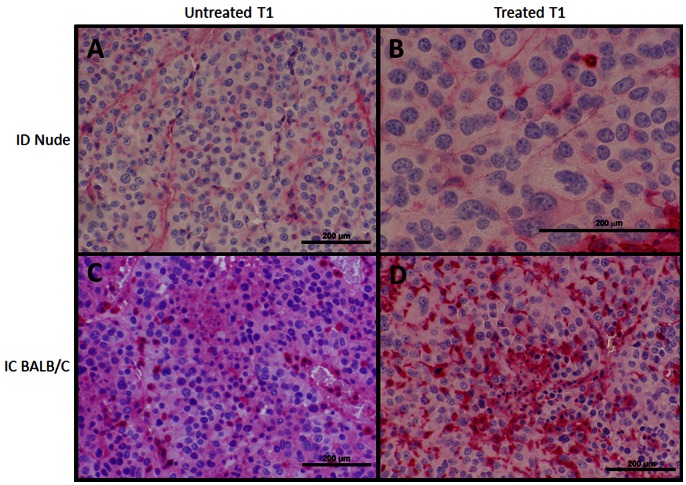
CD3^+^ Immunohistochemsistry of primary (T1) tumors. CD3^+^ staining, indicative for T-cell presence, performed for (A,C) untreated and (B,D) treated initial T1 tumors between (A,B) ID nude and (C,D) IC BALB/c mice. There is no notable difference observed in CD3^+^ infiltration for ID nude mice between (A) untreated and (B) treated tumors. For the IC BALB/c mice, a robust increase in CD3^+^ (T-cell) infiltration is observed in some treated tumors (D) relative to untreated T1 controls (C). Increased T-cell presence in treated T1 IC mice was also more robust than for both groups for nude mice (A,B). All scale bars 200 µm. Panels (A,C,D) 200x, panel (B) 400x magnification.

Microscopic examination of treatment effects were performed on formalin fixed, paraffin embedded tissue sections for the various treatment groups using Haematoxylin and Eosin (H&E) (Fig. 6) and CD3^+^ immunohistochemistry (Fig. 7). There was little immunocyte presence in untreated tumors and matrigel-only injections, with no appreciable difference between the ID nude and IC BALB/c mice. However, there was an inflammatory reaction present when the untreated tumors extended into muscle belly of the abdomen or there was scabbing present on the skin from the electric pulses, which occurred at the subcutaneous-cutaneous junction. In the treated primary (T1) and untreated rechallenge (T2) tumor inoculation sites of ID mice, there was relatively low immune response, with some neutrophils present, but no significant CD3^+^ T-cell infiltration, as expected, due to the ID strain’s athymic defect. Conversely, the IC mice showed a greater immune cell presence, with most immunocytes present in the treated T1 tumors occurring at the transition zone between viable and dead tumor. The CD3^+^ staining showed robust T-cell infiltration into the treated T1 tumors in some, but not all, IC mice compared to untreated T1 controls. Conversely, there is no notable difference in CD3^+^ cells between treated and untreated T1 tumors in the ID nude mice.

## Discussion

This study provides evidence supporting an improved irreversible electroporation localized tumor therapeutic response in an immunocompetent (IC) versus immunodeficient (ID) mouse tumor model. An additional protective effect against tumor rechallenge also is observed in IRE treated IC but not ID mice. While we provide empirical evidence to this improved and protective effect in IC mice based on tumor growth curves and histology, future work should examine the exact mechanisms inducing these enhanced and protective effects.

In the first portion of this investigation, we examine if tumor treatment response to IRE is greater in the presence of a complete, functioning immune system, versus immunodeficient athymic nude mice which lack functional T-cells. Studies have shown immunocyte presence in ablated regions and lymphatic drainage following healthy tissue ablations [8], [12], [13], but do not assess the potential improved patient response to IRE tumor therapy from these effects. IRE-induced tumor cell death was shown to occur independently from immunocyte interaction up to 24 hours after pulsing [33], but does not account for long-term effects, when the immune system has time to mount a supplemental attack on any remaining cancer cells. A recent study using an immunocompetent rat tumor model showed several changes in immunity following IRE tumor ablation, including changes in T-cell subset percentage, cytokine-positive splenocytes, and serum sIL-2R and IL-10 levels in peripheral blood [31]. In these studies examining immune response to IRE, discrete comparative evaluation of tumor response to these effects remained to be determined.

The treated IC group responded significantly better than the treated ID group, despite no inherent difference in tumor susceptibility between the two immune-status groups. Similar susceptibility is indicated by tumors reaching a treatable size for both groups within the same number of days, and similar tumor growth response to sham treatments (Fig. 2A–B). This suggests that although an immune response is not required for complete tumor regression [33], therapeutic response in immunocompetent patients may be better than predictions based on experimental studies using ID cancer models.

Because the ID Nu/Nu mouse model is an athymic-only immune deficiency, one mechanism for improved anti-tumor efficacy of IRE in the BALB/C IC mice may relate to adaptive immunity. Focal ablation causes local damage to the tissue that promotes an innate immune response, including inflammation and immunocyte infiltration, to resorb cellular debris. However, the functioning innate immunity in ID mice suggests that this alone is not as effective as both aspects of the immune system (innate and adaptive) in cooperation. This may relate to electroporation-induced tumor antigen release into the interstitium, initiating an immune response capable of locally improving tumor response and exhibiting a systemic protective effect. This immunostimulation would also occur for reversibly electroporated cells.

As noted in [20], the cause of cell death will play a significant role in determining the extent and effects of an immune response. The “danger theory” used to describe the immune recognition of “self” versus “non-self”, as well as dangerous [34], [35], describes 3 signals required for the generation of a cytotoxic T-cell response. These include recognition of the matching antigen for the T-cell receptor, a co-stimulatory signal between the antigen presenting cell (APC) and T-cell, and the third being a danger signal to activate the APC. According to the theory, if the first two signals are encountered without the third signal to activate the APC, such as IL-12, T-cell activation occurs in a strongly blunted fashion [36], and can even result in CD8^+^ T-cell peripheral tolerance [37].

The third danger signal can come in the form of exogenous markers for invading organisms, or endogenous signals based on the mechanism of cell death. Several studies have demonstrated that necrotic cells will lead to increased dendritic cell (DC) and macrophage activation [38], [39]. With necrosis, there is significant release of intracellular contents, such as pro-inflammatory cytokines, heat shock proteins, DNA and RNA recognized by Toll-like receptors, uric acid, or chromosomal protein HMGB1 (high mobility group box chromosomal protein 1) [40]; as well as signals based on tissue structural changes including fibrinogen, oligosaccharides of hyaluronan, heparin sulfate proteoglycan, and extradomain A (EDA)-containing fibronectin [20].

Apoptotic cells do not release their contents like necrotic cells, reducing the presence of danger signals, and some studies show that apoptotic cells actually mitigate immune recognition [41], [42], which may relate to peripheral tolerance when only self-antigen is presented [43], [44]. Where ECT typically relies on chemotherapeutics to induce apoptotic cell death [45], this may explain why some ECT studies cite the requirement of an additional immunostimulatory molecule, such as CpG-ODN or IL-2, to attain observable immunological enhancement to therapeutic outcome [26], [46]. Conversely, IRE focal ablation appears to kill cells through several mechanisms. While there is evidence for IRE to induce apoptosis in treatment regions [12], [47], particularly at lower lethal electric fields [48]; it has also been shown to cause a strong and immediate necrotic effect as a major mechanism of cell death [8], [11], [13], [33]. It is possible that IREs necrotic cell death modality alone provides appropriate immuno-stimulatory signals required for a robust immune response to enhance the therapeutic outcome. This is one possible explanation why IRE attained improved local anti-tumor efficacy in IC versus ID mice, and inhibition of re-challenge tumors, without adjuvants.

It should be noted that the effect regarding apoptotic cells is not entirely clear, and some studies have shown apoptotic cells to produce a superior immune effect relative to necrotic cells [49], [50]. Such an effect may result from stimulation of an anti-tumor immune response induced by secondary necrosis of uncleared apoptotic cells [20]. This is consistent with the observation that therapeutic electroporation techniques that primarily induce apoptotic cell death, including ECT and nanosecond pulsed electric fields [45], [51], may benefit from an immunological enhancement that is sufficient to mitigate tumor re-challenge [52]. IREs apoptotic mechanisms may also exhibit these effects.

The IC murine model showed slowed or completely inhibited growth of a rechallenge inoculation with the same cancer cell line. It should be noted that too few control IC and both groups of ID mice survived the initial tumor burden remaining less than 18 mm in diameter long enough to examine if this effect would occur for them as well. However, the observed response in the rechallenged IRE treated IC mice supports the possibility for future studies to investigate the promotion of a systemic response capable of targeting distant regions of cancerous cells undetectable by modern techniques, both experimentally and clinically, when using IRE to treat the visible regions of tumor. This capability remains speculative, and would likely benefit from the inclusion of adjuvant immunostimulants to fully exploit such a potential. Such techniques may be particularly beneficial when using immunostimulants that encourage adaptive immunity.

The tumor immune response observed in this study may derive itself from IREs non-thermal method of causing cell death. By remaining below temperatures that cause protein coagulation and molecular denaturation, dead electroporated cells will maintain identifying molecular antigens that may be used by antigen-presenting cells to activate systemic immunity, when presented in conjunction with additional required immune co-stimulating molecules. Further, in addition to evidence for IRE-induced tumor cell apoptosis, the evidence for IRE to cause immediate necrosis of the ablated region may present the body’s immune system with all three signals required to incite a tumor-specific adaptive immune response. IRE ablation promotes near-immediate edema and an inflammatory response, rapidly attracting lymphocytes to the treated region, which may acquire antigens as well as kill remaining cancer cells that survived the IRE electric pulses. These aspects of improved IRE local therapeutic response may also further benefit from the addition of adjuvant immunostimulants, which should be examined in future work.

### Conclusion

This investigation examined the potential improved anti-tumor response in immunocompetent versus immunodeficient mice from IRE focal ablation tumor therapy. Subcutaneous tumors in immunocompetent and immunodeficient mice were treated with a low IRE pulsing protocol designed to avoid complete regressions in immunodeficient mice. Tumor response in the treated immunocompetent mice was significantly stronger than the treated immunodeficient mice, or either of the sham controls from each mouse strain. Rechallenge with the same cancer cell line 18 days after IRE resulted in limited, if any, second tumor growth in the treated immunocompetent mice. Histology showed robust CD3^+^ cell infiltration in some, but not all, treated immunocompetent BALB/C mouse tumors; without significant infiltration in untreated controls or either treatment group of immunodeficient Nu/Nu mice. This study provides evidence for enhancement of local and distant therapeutic outcome in immunocompetent cancer patients following IRE treatment.
